# Sensing and Automation Technologies for Ornamental Nursery Crop Production: Current Status and Future Prospects

**DOI:** 10.3390/s23041818

**Published:** 2023-02-06

**Authors:** Md Sultan Mahmud, Azlan Zahid, Anup Kumar Das

**Affiliations:** 1 Department of Agricultural and Environmental Sciences, Tennessee State University, Nashville, TN 37209, USA; 2 Otis L. Floyd Nursery Research Center, Tennessee State University, McMinnville, TN 37110, USA; 3 Department of Biological and Agricultural Engineering, Texas A&M AgriLife Research, Texas A&M University System, Dallas, TX 75252, USA; 4 Department of Agricultural and Biosystems Engineering, North Dakota State University, Fargo, ND 58102, USA

**Keywords:** agricultural mechanization, artificial intelligence, computer vision, digital agriculture, internet-of-things, plant biometrics, smart irrigation, smart spraying, stress detection

## Abstract

The ornamental crop industry is an important contributor to the economy in the United States. The industry has been facing challenges due to continuously increasing labor and agricultural input costs. Sensing and automation technologies have been introduced to reduce labor requirements and to ensure efficient management operations. This article reviews current sensing and automation technologies used for ornamental nursery crop production and highlights prospective technologies that can be applied for future applications. Applications of sensors, computer vision, artificial intelligence (AI), machine learning (ML), Internet-of-Things (IoT), and robotic technologies are reviewed. Some advanced technologies, including 3D cameras, enhanced deep learning models, edge computing, radio-frequency identification (RFID), and integrated robotics used for other cropping systems, are also discussed as potential prospects. This review concludes that advanced sensing, AI and robotic technologies are critically needed for the nursery crop industry. Adapting these current and future innovative technologies will benefit growers working towards sustainable ornamental nursery crop production.

## 1. Introduction

The nursery and greenhouse industry contributes nearly $14 billion in annual sales to the U.S. economy [1]. This industry produces more than 2000 ornamental plant species, covering most of the U.S.’ ornamental plants [2]. Nurseries are, in general, open-air operations where plants grow in the ground or in containers [3]. Greenhouses are typically enclosed environments where growth conditions (e.g., lighting, temperature, humidity, and irrigation) can be controlled [4]. Rapidly increasing production cost due to the increased labor expense, difficulty in obtaining skilled labor, and inappropriate application of agricultural resources are rising concerns for the ornamental industry [5,6]. Operations such as planting, growing, and harvesting nursery crops are heavily dependent on labor. These operations account for 43% of total production expenses [7]. It is becoming increasingly difficult for the industry to obtain such labor, especially the skilled workforce required to grow ornamental crops [8]. Conventional practices apply agricultural resources (such as water, nutrients, fertilizers, and pesticides) excessively and inefficiently, increasing production costs. These conventional approaches not only increase the production cost but are also responsible for contaminating the environment and the ecosystem. The industry must look for alternative solutions, such as automated crop management technologies, to reduce labor needs and ensure the efficient use of crop production resources.

In the current decade, sensing and automation technologies have been continually increasing their impact on different crop management operations [9,10,11,12,13]. These technologies are categorized into two groups: ground-based and aerial-based. Ground-based crop harvesting technologies have been tested on various crops, including sweet pepper [14], lettuce [15], tomato [16], strawberries [11], apples [9], and cherries [17]. Ground-based technologies have also been explored widely in automatic disease detection in different crops, such as: powdery mildew on strawberry leaves [18]; leaf blotch, stripe rust, powdery mildew, leaf rust, black chaff, and smut on wheat leaves [19]; Alternaria leaf spot, brown spot, mosaic, grey spot and rust on apple leaves [20]; and anthracnose, brown spot, mites, black rot, downy mildew, and leaf blight on grape leaves [10]. Recent evolutions in unmanned aerial vehicles (UAVs) show the potential of using them in different agricultural operations, thereby consuming less time than ground-based systems [12]. Until now, UAVs used for agriculture have been limited to only remote sensing applications, due to limited payload capacity and battery life. UAVs have been used in various crop management applications, including automatic canker disease monitoring in citrus [21], weed detection in wheat and oat fields [22], detecting and mapping tree seedlings and individual plants [23,24], and yield estimation in cotton [25]. However, the success of sensing and automation technologies largely depends on the types of sensors used to acquire crop data and the processing algorithms used to extract valuable information.

Various sensors, such as soil moisture, temperature and humidity sensors, cameras (color, spectral, and infrared), together with computer algorithms are used to develop smart technologies for agricultural applications [5,18,21,26]. A prototype irrigation controller system was developed using nine soil moisture sensors on an IoT platform to automatically manage water application in crops [26]. You et al. [27] used an RGB-D camera system to develop an autonomous robot for pruning branches of sweet cherry trees. It should be noted that RGB-D cameras offer four channels (i.e., red, green, blue and depth) that were required to estimate the size of branches (by depth channel) to decide which ones need to prune. Abdulridha et al. [21] detected citrus disease at an early stage using a hyperspectral camera. Other cameras may not be suitable for detecting a particular disease at the asymptomatic stage. Liu et al. [28] used enhanced generative adversarial networks (GANs) to augment their data for grape leaf disease detection; other machine-learning models were not considered because of the requirement for a deeper network.

In conclusion, identifying appropriate sensors and developing algorithms are necessary tasks that depend mainly on crop and soil characteristics and operational needs. In most cases, one automated technology is specific to one particular operation in a specific crop. Therefore, evaluating sensor and algorithm performances for different crops in a certain industry provide insights for choosing them generally, while developing technology for a particular production operation. Although the ornamental crop industry is in the initial phase of developing sensing and automation technologies, an overview of currently available technologies and prospects of advanced technologies utilized for other crop industries (for agronomic crops and tree fruits industry) will be helpful for future technology developments.

### 1.1. Scope of the Study

A few of the available reviews for ornamental crops mainly reviewed water management technologies and barriers to technology adoption [6,29]. Lea-Cox et al. [29] studied the economic benefit, current and future challenges, and support issues of using wireless sensor networks (WSNs) for water management of ornamental crops. Rihn et al. [6] reviewed factors correlated with the nursery industry’s propensity to use automation and mechanization. Their study also discussed the barriers to adoption for currently available automated technologies. This review aims to cover available sensing and automation technologies used for ornamental crop production operations, along with the prospects of using some advanced technologies (used in other crop industries) that can be beneficial to this industry. To the author’s knowledge, this is the first review article that broadly discusses sensing and automation technologies for ornamental crops.

### 1.2. Paper Organization

This review aims to discuss the status and challenges of sensing and automation technologies for the ornamental crop industry. The organization of this article is as follows: Section 2 presents an overview of sensing and automation technologies used for ornamental crops. In Section 3, advanced technologies used for other cropping systems are discussed that could be valuable for developing future technologies for ornamental crops. Finally, Section 4 summarizes the overall discussion and conclusion of the article.

## 2. Sensing and Automation Technologies for Ornamental Crops

**Figure 1:**
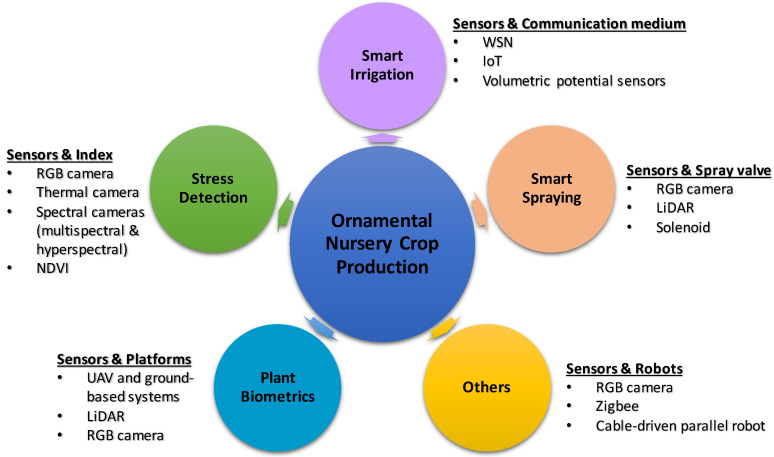
Areas where sensing and automation technologies are used for ornamental crop production.

Sensing and automation technologies are used in different operations relating to ornamental nursery crop production. The major operations are smart irrigation, plant stress detection, smart or variable-rate spraying, and plant biometrics measurements (Figure 1). This section presents detailed reviews of the currently applied sensing and automation technologies for those operations. The technologies have also been used in a few other areas and represented as other significant works.

### 2.1. Smart Irrigation

**Figure 2:**
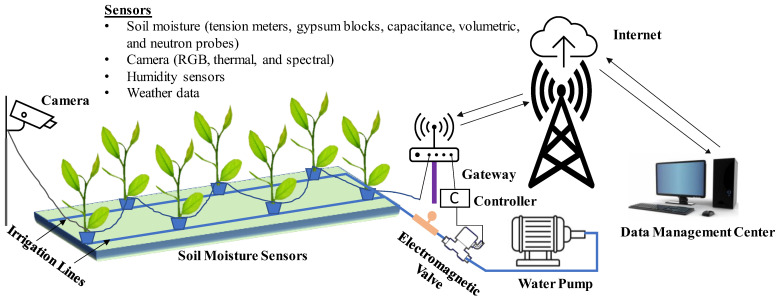
A schematic of an IoT-based smart irrigation system for water management in a container-based nursery.

Smart or precision irrigation technology determines the water requirement of crops using set-point control (using soil moisture data) or model-based control (using crop and environmental data) to maximize irrigation efficiency [4,29]. It helps reduce excessive water application while maintaining crop growth and development. Sensors-based irrigation technologies have been tested in different nurseries, including greenhouse, container, pot-in-pot, and field nurseries [30,31,32,33,34]. A schematic diagram of a smart irrigation system is presented in Figure 2.

Table 1 presents different sensor applications for automatic irrigation management in different nurseries. Wireless sensor networks (WSNs) were used to control irrigation water flow in three container-based nurseries [32]. Experiments were conducted in two phases: first, EM50R nodes with EC-5 sensors were used to monitor soil moisture; and second, nR5 nodes were used to monitor and control irrigation. The WSNs-based technology reduced water use by about 20% to 25%. Kim et al. [35] tested soil moisture and EC sensors to monitor and automatically implement irrigation protocols. Substrate moisture data were measured to reduce water usage of hydrangea by as much as 83%. Coates et al. [36] used a VH400 (Vegetronix, Sandy, UT, USA) sensor to monitor soil water content in container nurseries where pots contain hydrangea plants. Even though the VH400 sensor costs half as much as standard EC-5 sensors, the authors concluded the VH400 was unsuitable for nursery crop monitoring because its output varied by up to 29%. This type of sensor (VH400) shows a high sensitivity of ~34 mV rather than ~5 mV using EC-5 per % volumetric water content. Lea-Cox et al. [31] used a hybrid system consisting of a 12-node CMU network (developed by Carnegie Mellon University, United States) and Decagon Ech20 moisture sensors (Decagon Devices Inc., Pullman, WA, USA) to control water applications in real-time in a container nursery. The system was also tested in a greenhouse where a six-node CMU network was used. The results reported that both networks performed well, but encountered some networking challenges at remote sites. The authors noted the CMU network node is less costly than the commercial Decagon Ech20 sensor, but showed similar performance. Wheeler et al. [34] also tested a smart irrigation system in a container nursery and greenhouse. They used Decagon soil moisture sensors along with an nR5 wireless node to control irrigation. The study reported a water use reduction of approximately 50% when compared to grower-controlled irrigation. The same sensor system was trialed previously by Wheeler et al. [5] in a floriculture greenhouse.

The WSNs are also used in pot-in-pot nurseries. Belayneh et al. [37] used this technology to control irrigation in dogwood (planted in 15-gal containers) and red maple (planted in 30-gal containers) nurseries. The EM50R nodes were used to monitor data from soil moisture, and environmental sensors and nR5 nodes were used for irrigation control. Volumetric water content-based sensors were utilized for monitoring soil moisture. The sensors were inserted at a 6-inch depth for dogwood and at 6 and 12 inches depth for red maple. The results showed that the WSNs-based irrigation method reduced water usage by ~34% and ~63% for red maple and dogwood, respectively. Lea-Cox and Belayneh [38] developed a smart battery-operated nR5 wireless sensor node using a series of soil moisture and environmental sensors to irrigate dogwood and red maple nursery blocks. The study reduced daily water application by about 62.9%. The authors concluded that this sensor-based irrigation technology resulted in nearly a three-fold increase in the efficiency of water without reducing the quality or growth of trees.

Internet-of-Things (IoT)-based smart irrigation systems have also been used for ornamental crop production. Banda-Chávez et al. [39] developed an IoT-based sensor network to activate the irrigation system to irrigate ornamental plant using an IoT platform and soil moisture sensors (YL-69). In addition, Beeson and Brooks [40] used an evapotranspiration (ETo) model-based smart irrigation system for wax-leaf privet. The study reported that this model-based irrigation system could reduce water application by about 22.22% annually, compared to the traditional overhead irrigation method. Although a limited number of studies have reported on the IoT-based automatic irrigation systems used for the ornamental industry, trends and current successes of this technology for other crop industries show promising potential for ornamental crop production.

Although studies have reported the potential of using sensors-based technology for irrigation management, many factors impede this technology’s efficacy. Sensor-to-sensor variability in a particular environment could be one of them. The greatest variability among sensor readings occurred at volumetric water content levels just below the water-holding capacity of the substrate. Therefore, finding sensor-to-sensor variability in a particular nursery condition can greatly increase confidence in the data. Sensor positioning is another important factor that directly affects efficacy. Accurate positioning is needed in nursery conditions, particularly when measuring soil moisture content in container production. Sensors need to be placed in that part of the root zone where active water uptake occurs. Determination of optimal sensor numbers is another factor in specifying sensors for a nursery environment. The optimal number of sensors for a particular nursery depends primarily on the accuracy and repeatability of the sensors, variation among sensors, spatial variability of the nursery environment, and cost.

**Table 1:** Summary of studies reported for smart nursery irrigation.

Crop	Nursery Types	Soil Sensor Types	Water Saving	References
Ornamentals	Container	Capacitance-based (WSNs)	20% to 25%	Chappell et al. [32]
Hydrangea	Container	Capacitance-based (WSNs)	Not Reported	Coates et al. [36]
Red Maple and Cherokee Princess	Container and Greenhouse	Matric potential and capacitance sensors (WSNs)	Not Reported	Lea-Cox et al. [31]
Hydrangea	Container	Electrical conductivity (WSNs)	As much as 83%	Kim et al. [35]
Woody Ornamental Plants: Oakleaf Hydrangea, Japanese Andromeda, Catawba Rosebay and Mountain Laurel	Container and Greenhouse	Capacitance-based (WSNs)	50%	Wheeler et al. [34]
Dogwood and Red Maple	Pot-in-pot	Capacitance-based (WSNs)	34% to 63%	Belayneh et al. [37]
Dogwood and Red Maple	Pot-in-pot	Capacitance-based (WSNs)	62.9%	Lea-Cox and Belayneh [38]
Ornamental plants	Pots in indoor	Capacitance-based (IoT)	Not Reported	Banda-Chávez et al. [39]

### 2.2. Plant Stress Detection

**Figure 3:**
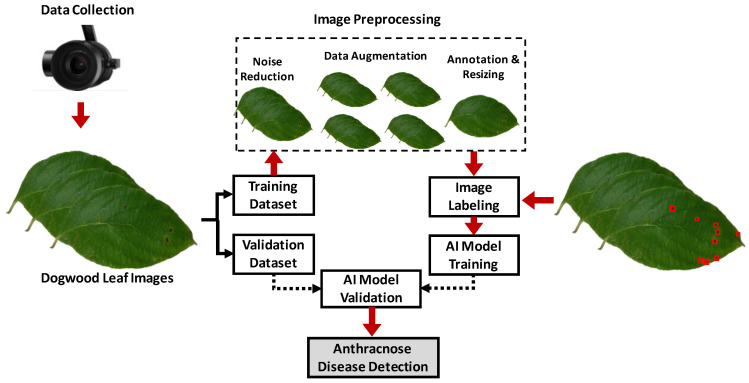
A schematic of a computer-vision-guided dogwood anthracnose leaf disease detection procedure.

Detection of stresses such as drought, disease infection, and pest pressure, recognizes unfavorable condition or substance that affects the growth, development or production of plants or crops using sensors and advanced technologies [41]. This detection helps growers to identify problems and take preventive actions before stresses significantly damage plants or crops. Two types of stresses have been identified in ornamental crop production: abiotic plant stress and biotic plant stress. Abiotic plant stress includes drought, nutrient deficiency, salinity problems, floods, etc., while biotic stress refers to damage caused by fungi, bacteria, insects, or weeds. Sensors, including RGB, thermal, and spectral, have been utilized to monitor stresses in ornamental crop production [42,43,44,45]. A schematic diagram of the sensor-based automatic crop disease detection procedure is presented in Figure 3.

Table 2 represents different ornamental plant disease detection using advanced sensing technologies. Red-green-blue (RGB) imaging sensors with a spectrum range of 400–700 nm (visible range) are used to monitor ornamental plant stresses due to their affordability and application in other cropping systems. Velázquez-López et al. [42] developed an image processing-based powdery mildew disease detection system for rose plants by using the Open CV library. The system detected powdery mildew by converting RGB images to hue, saturation, and value (HSV) color space and achieved the highest disease region matching of 93.2% by segmenting with V channel using close captured images (captured at 10 cm from the rose canopies). Although this study achieved good performance with the traditional image segmentation method, the performance would not have been the same if the image capturing conditions had changed. This is considered a major limitation, especially for real-time disease detection, where multiple diseases would be present. Nuanmeesri [46] advanced the image processing technique from traditional image segmentation to deep learning-based detection in order to identify up to 15 different diseases. A hybrid deep learning model built by fusing convolutional neural networks (CNNs) and a support vector machine (SVM) were used. Researchers also tested the image registration approach of two imaging media for ornamental crop disease detection. Minaei et al. [45] registered RGB and thermal images to detect powdery mildew and gray mold disease on roses for developing a site-specific spraying system. A few studies have compared RGB imaging with spectral imaging for tulip disease detection [43,47]. The results reported that a spectral imaging system achieved better detection accuracies than RGB imaging while detecting tulip breaking virus (TBV).

Hyperspectral imaging is a powerful tool that uses imaging and spectroscopy for detecting stresses at the early stage, gathering and processing feature information from a wide spectrum of light. Researchers have used hyperspectral sensors for ornamental crops, but mainly in laboratory applications due to their vulnerability in real-time field applications [43]. Polder et al. [48] identified Botrytis infected Cyclamen plants with selected features (bands) of 497, 635, 744, 839, 604, 728, 542, and 467 nm in a controlled greenhouse environment. Poona and Ismail [44] selected wavebands located across VIS, red edge, NIR, and SWIR regions to detect *Fusarium circinatum* infection in Pinus radiata seedlings at the asymptomatic stage. The study concluded that random forest (RF) is a good machine learning (ML) classifier to discriminate disease infection from spectral bands. Heim et al. [49] also used RF to differentiate myrtle rust-infected lemon myrtle plants and achieved an overall accuracy of 90%. The spectral wavebands (545, 555, 1505, and 2195 nm) were selected for discrimination. Considering hyperspectral systems’ slow data processing and expense, some studies have tried to find an alternative to hyperspectral imaging. A few studies have used the multispectral imaging system instead because of its faster data processing ability. Polder et al. [43] used an RGB-NIR-based multispectral system (range 500–750 nm) to detect TBV disease in tulips and achieved a classification accuracy of 92%. They employed a linear discriminant classifier along with R, G, B, and NIR features to segment the plant and the soil. The author used features such the fraction of red pixels, mean normalized red value, mean normalized green value, and ratio of contour pixels of spots to classify disease in tulips. Pethybridge et al. [50] assessed ray blight disease (caused by *Phoma ligulicola*) intensity using a hand-held multispectral radiometer with 485, 560, 660, 830, and 1650 nm spectral band sensors. The study used vegetation indices, including normalized difference vegetative index (NDVI), green normalized difference vegetative index (GNDVI), difference vegetative index, and renormalized difference vegetative index to assess ray blight disease.

Thermal imaging has also been tested for stress detection in ornamental plants, a technique which depicts the spatial distribution of temperature differences in a captured scene by converting infrared (IR) radiation into visible images. Jafari et al. [51] classified asymptomatic powdery mildew and gray mold disease on roses by fusing thermal images with visible-range captured images. Valuable thermal features were extracted, and artificial neural networks (ANN) and SVM were used to classify healthy and disease-infected rose plants. The thermal features include maximum, minimum, median, mode, standard deviation, maximum difference in temperature, skewness, kurtosis, sum of squared errors, and so on. Studies have been conducted for disease stress detection using thermal imaging; however, this type of sensing is more practical for water stress detection. Before conducting the above experiment, Jafari et al. [52] attempted to classify *Botrytis cinerea* infection on rose using thermal spectra and radial-basis neural networks. Buitrago et al. [53] analyzed the infrared spectra of plants for water stress detection and concluded that spectral changes in plant regions had a direct connection with the microstructure and biochemistry of leaves.

Stress detection technologies are widely used in other crop industries, especially for agronomic crops (such as corn and soybean) and tree fruits (such as apple and citrus), but very few experiments have been conducted for ornamental crops (mostly in the floriculture industry). Very limited research, almost no studies, have been conducted for the woody ornamental industry. A few studies have been conducted to detect stress using RGB sensors because RGB cameras do not require deep technical knowledge to operate or use. Spectral sensors are necessary to detect stress at an asymptomatic or early stage. Spectral sensors have a huge potential for the ornamental industry, but not much progress has been previously reported. Currently, UAVs are very popular for crop stress detection and monitoring, but the applications of these systems are also very limited for the ornamental crop industry. De Castro et al. [54] used a UAV system to detect water stress in Cornus, Hydrangea, Spiraea, Buddleia and Physocarpus, and the results of this study show promise. The ornamental industry can benefit from using UAV-based sensing technologies for the timely detection and monitoring of stresses to enhance crop production.

**Table 2:** Summary of studies reported for plant stress detection.

Crop	Stress Type	Imaging Type	Processing Method	Accuracies	References
Rose	Powdery mildew	RGB (a video camera: Everio)	Images were converted to HSV, and then segmentation performed to extract the disease region	Highest 93.2% of disease region matching	Velázquez-López et al. [42]
Rose	Fifteen different rose diseases	Color images downloaded from the Google search engine and ChromeDriver	A hybrid deep learning model (CNNs with SVM)	90.26% accuracy, 90.59% precision, 92.44% recall, and 91.50% F1-score	Nuanmeesri [46]
Rose	Powdery mildew and gray mold	RGB (Canon 550D Kiss X4); Thermal camera (ITI-P400)	Image registration of visible and thermal images and then segmentation to segment diseased area	Not reported	Minaei et al. [45]
Tulip	Tulip breaking virus	RGB (Nikon D70 with a NIKON 18–70 mm zoom lens); Spectral camera (Specim, spectrum from 430 to 900 nm with a resolution of 4.5 nm)	Spatial information was extracted after segmentation, and then Fisher’s linear discriminant analysis (LDA) used for the detection	Best results of 9, 18 and 29% detection error were achieved for Barcelona, Monte Carlo, Yokohama tulip variety, respective using the spectral camera	Polder et al. [47]
Tulip	Tulip breaking virus	RGB (Prosilica GC2450 and GC2450); RGB-NIR multispectral (JAI AD120GE); Multispectral (using six-band filter wheel, range 500-750 nm)	Plant segmented by thresholding the excessive-green image ((2G–R–B) > 0) and then LDA for TBV classification	92% of TBV-diseased plants were accurately classified using RGB-NIR multispectral system	Polder et al. [43]
Cyclamen	Botrytis	Hyperspectral imaging (400–1000 nm)	Selected most discriminating wavelengths and then applied LDA	90% of pixels were classified correctly	Polder et al. [48]
*Pinus radiata* seedlings	Pitch canker disease (*F. circinatum* infection)	Hyperspectral imaging (600–2500 nm)	Wavebands were selected using the Boruta algorithm, and then Random forests were used for discriminating infected seedlings	0.82 and 0.84 KHAT values for healthy-infected and infected damaged discrimination, respectively	Poona and Ismail [44]
Lemon myrtle	Myrtle rust	Hyperspectral imaging (350–2500 nm)	Four wavebands were chosen, and RF was applied for discrimination	90% of overall accuracy	Heim et al. [49]
Pyrethrum	Ray blight disease	Multispectral radiometer	Reflectance was measured, and data were analyzed using regression analysis	Not reported	Pethybridge et al. [50]
Rose	Powdery mildew and gray mold	Infrared thermal camera (ITI-P400)	Image registration and then segmentation were performed to extract features, and finally, neuro-fuzzy classifiers were used for classification	92.3% and 92.59% estimation rates were achieved for powdery mildew and gray mold, respectively	Jafari et al. [51]
Rose	*Botrytis cinerea* infection	Infrared thermal camera (ITI-P400)	Analyzed extracted thermal features with radial-basis neural networks	96.4% correct estimation rate	Jafari et al. [52]

### 2.3. Smart Spraying

**Figure 4:**
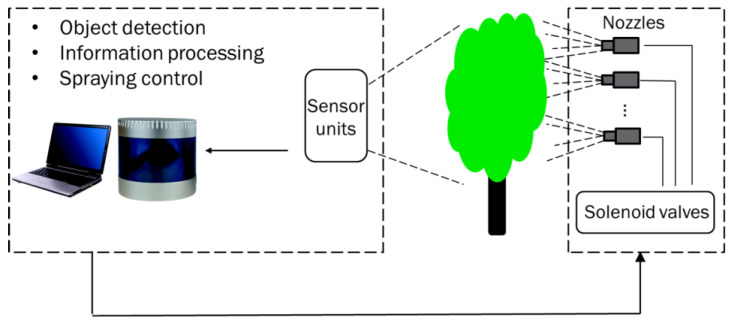
A schematic of a light detection and ranging (LiDAR)-guided variable-rate spraying system.

Management of different pests and diseases is essential to ensure high quality ornamental nursery crop production meeting the market’s requirements [55]. Traditional management techniques include pruning the infected branches, removing dead or infected plants, monitoring diseases, trapping insects, growing pest-resistant cultivars, and pesticide applications [56]. Foliar pesticide application is the most effective method for preventing pest infestations and ensuring healthy and unblemished nursery plants [57]. In the United States, the greenhouse and nursery industries use about 1.3 million kg of pesticides every year, saving billions worth of crops [58]. Conventionally, radial air-assisted sprayers are the most used spray equipment for pesticide application in ornamental nurseries [59]. These sprayers apply pesticides to the entire field regardless of the plant structure, plant growth stage, and absence of plants in rows, thus, resulting in under- or over-spraying [60] as well as contaminating the environment, wasting pesticides, and increasing production cost [61]. This problem is more critical for the nursery industry, as there is great diversity in canopy structures and densities found in nursery crops. In field nursery production, it is a common practice that trees of different ages and cultivars are planted in the same row. The traditional sprayers cannot adjust sprayer settings to match the target tree requirements, reducing application efficiency. One way to improve spraying efficiency is to use sensing technologies to identify target trees for precise spraying applications, also referred to as smart/variable-rate-intelligent spraying (Figure 4).

Smart spraying is defined as the precise application of pesticides, performed by controlling the spray output of each nozzle based on the presence, structure, and canopy density of plants as obtained from sensors such as ultrasound, laser, and cameras [18]. In recent years, significant research has been conducted to develop smart spraying systems for the nursery industry. Different sensors, such as ultrasonic and laser, have been utilized to measure the canopy parameters for intelligent spraying in nursery crops. The summary of the reviewed studies is presented in Table 3. The initial efforts for smart nursery spraying were reported back in 2010 by a team of scientists from the United States [62]. The authors developed two precision sprayer prototypes: a hydraulic boom sprayer with an ultrasonic sensor for small narrow trees such as liners and an air-assisted sprayer with a laser scanner for other ornamental nursery species. The authors compared the spray consumption between a sensor-based sprayer and a conventional air blast sprayer at three growing stages and four travel speeds (3.2, 4.8, 6.4, and 8.0 km/h). The sensor-based air-assisted sprayer applied 70%, 66%, and 52% fewer chemicals at different growth stages than conventional spraying. The results also reported a uniform spray deposit and coverage regardless of changes in the canopy size and travel speed. Jeon and Zhu [63] developed an ultrasonic-sensed real-time variable-rate vertical boom sprayer for nursery liners. The sprayer consisted of two booms with five pairs of equally spaced nozzles, with the ultrasonic sensor mounted 0.35 m ahead of the nozzles. Field tests were conducted for six different liner species at travel speeds from 3.2 to 8.0 km/h. The spray nozzles were triggered successfully from 4.5 to 12.5 cm ahead of the target, and the effects of travel speed on mean spray coverage and deposit were insignificant. Following this work, a study for the same precision sprayer was reported for performance evaluation based on spray coverage, deposit, and droplet density compared to conventional ones for all six-liner cultivars [64]. The reported results suggest that the spray coverage, deposit, and droplet density were lower in the sensor-based sprayer, and the spray volume was reduced by 86.4% compared to the conventional sprayer. 

Laser sensing is another technology used for precision spraying for many tree crops. A few studies have been reported that utilize laser scanning for smart spraying applications in nurseries. Chen et al. [57] developed a variable-rate air-assisted sprayer using a laser scanner. The authors reported that the spray coverage differences inside the canopies were not statistically significant at 3.2 and 6.4 km/h travel speeds. Liu et al. [65] used a laser scanner to develop an intelligent variable-rate air-assisted sprayer and tested the system in a commercial nursery and grapevine orchard. The authors reported that the new sprayer reduced chemical usage by more than 50% compared to the conventional sprayer at a travel speed of 3.2 to 8.0 km/h. Shen et al. [66] developed an air-assisted laser-guided sprayer for Japanese maple nursery trees. The new sprayer consisted of a 270° radial-range laser scanner, embedded controller, and pulse-width-modulated (PWM) nozzles. The authors reported an accurate measurement of different trees and control of nozzles to match trees independently. The spray usage was reduced by 12 to 43%, compared to the conventional spraying. In addition, a few studies have been reported for field validation of precision sprayers to control different diseases. Zhu et al. [59] validated the laser-guided air-assisted sprayer and reported a chemical saving of about 36% and 30% in the Prairifire crabapple and Honey locust nurseries, respectively. Chen et al. [67] also conducted a performance comparison of laser-guided air-assisted sprayers with conventional sprayers in commercial nurseries with different test plants. The author reported 56% and 52% chemical savings for two nurseries. Similarly, a few other studies have compared the performance of smart laser-guided sprayers with conventional sprayers and reported promising results for effective disease control in different nursery crops [61,68].

**Table 3:** Summary of studies reported for smart nursery spraying.

Crops	Nursery Types	Sprayer and Sensor Type	Performance	References
Multiple ornamental tree species	Field nursery	Two sprayers: Vertical boom with an ultrasonic sensor; Air assisted sprayer with a laser sensor	Chemical usage was reduced by 70%, 66%, and 52% at different growth stages of the target trees; achieved uniform spray deposits at all tested travel speeds	Zhu et al. [62]
Multiple ornamental tree species	Field nursery liners	Spray boom with ultrasonic sensor	The mean spray deposit was 0.72–0.90 μL/cm^2^; the mean spray coverage was 12–14.7%	Jeon and Zhu [63]
Multiple ornamental tree species	Field nursery liners	Spray boom with ultrasonic sensor	Spray volume was reduced by 86.4%; lower spray deposit and droplet density	Jeon et al. [64]
Tsuga canadensis Thuja occidentalis	Container-grown	Laser scanner air-assisted sprayer	Spray coverage differences were not significantly different	Chen et al. [57]
Ornamental nursery and grapevine	Field nursery	Laser scanner air-assisted sprayer	Chemical usage reduced by 50% at a travel speed of 3.2 to 8.0 km/h	Liu et al. [65]
Japanese maple	Field nursery	Laser-guided air-assisted sprayer	Spray savings of 12 to 43%	Shen et al. [66]
Prairifire crabapple Honey locust	Field nursery; pot-in-pot	Laser-guided air-assisted sprayer	Chemical savings of 36% and 30% in the Prairifire crabapple and Honey locust nurseries, respectively	Zhu et al. [59]
Multiple ornamental tree species	Field nursery	Laser-guided air-assisted sprayer	Chemical savings of 56% and 52% for two nurseries	Chen et al. [67]

Smart spraying for nursery crops using different sensing technologies, mainly ultrasonic and laser, has been reported in the last decade. Ultrasonic and laser sensors were integrated with conventional sprayers to detect the target (e.g., canopies). Although ultrasonic sensor-based sprayers exhibit significant chemical savings, their accuracy varies with temperature, humidity, and detection distance [57]. On the other hand, laser sensors are less influenced by weather conditions when detecting and measuring target characteristics [69]. Moreover, the nursery industry encounters several unique challenges, such as the lack of crop uniformity, varying shapes, sizes, growth patterns, and harvest schedules. Most existing sprayers have been developed for the orchard environment [59]; modifications may be required to make them usable for ornamental nursery crop production. Another challenge for the ornamental industry is its high aesthetic thresholds allowing for no visible infections. Thus, efforts are required to develop a smart spraying system based on the requirements of the nursery industry.

### 2.4. Plant Biometrics and Identification

Information on plant physiology and responses to biotic/abiotic stresses are critical to determine the management practices required to improve productivity and sustainability in the nursery industry. Plant biometry (e.g., structural information) can assist in understanding the plant’s growth differences in diverse environments [70]. Cultivar identification of nursery plants is also important for breeding, reproduction, and cultivation [71]. Plant biometry is a classification system that distinguishes a plant by defining its authenticity using physiological characteristics. The defined biometric for an individual plant should be universal, distinctive, permanent, and collectible [72]. 

**Figure 5:**
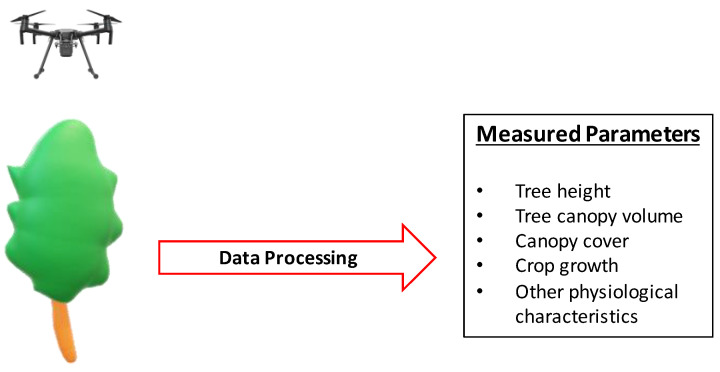
A schematic of a UAV-based tree canopy characteristics measurement system.

Plant identification, inspection, and a precise count of each cultivar’s number and size distribution are essential for nursery management and efficiently marketing the trees [73] (Figure 5).

Different sensors, including cameras and LiDAR, have been utilized for nursery plant biometrics. The summary of the reviewed studies is presented in Table 4. The research for nursery plant identification using camera imaging systems started in the 1990s. Shearer and Holmes [74] used a camera vision system to identify tree species in the nursery. The study used color co-occurrence matrices derived from intensity, saturation, and hue to identify seven common containerized nursery plants. A total of 33 texture features were used for the analysis, and the reported classification accuracy was 91%. She et al. [75] developed a high-resolution imaging system to classify containerized Perennial peanut and Fire chief arborvitae plants for counting. he authors found that the classification accuracy of plants with flowers was higher (97%) than those without flowers (96%). Leiva et al. [76] developed an unmanned aircraft system (UAS)-based imaging system for counting container-grown Fire Chief arborvitae. The author developed a custom counting algorithm and tested it on different backgrounds, including gravel and black fabric. The reported results indicated counting errors of 8% and 2% for gravel and black fabric backgrounds, respectively. 

In another study, the authors used a depth camera for height measurements of nursery plants [77]. The authors implemented Ghostnet–YoloV4 Network for measuring height and counting different nursery plants, including spruce, Mongolian scotch pine, and Manchurian ash. They achieved an accuracy of more than 92% for measurement and counting. Gini et al. [78] used a UAS-based multispectral imaging system to classify eleven nursery plant species. The author implemented multiple grey level co-occurrence matrix algorithms to perform textural analysis of acquired images. A principal component analysis was used after feature extraction, achieving a classification accuracy of 87% for the selected plants. Likewise, a few studies have reported the application of LiDAR sensors to identify nursery plants. Weiss et al. [79] developed a method for identifying nursery plant species using a LiDAR sensor and supervised machine learning. The author used multiple machine learning classifiers and 83 features to identify six containerized nursery plant species, and achieved an accuracy of more than 98%.

Similarly, LiDAR and light curtain sensors were used to develop a stem detection and classification system for almond nursery plants [73]. The authors developed a custom segmentation and thresholding algorithm, and the reported detection accuracies with the LiDAR and light curtain sensors were 95.7% and 99.48%, respectively. The success rates for dead/alive plant detection for the LiDAR and light curtain sensors were 93.75% and 94.16%, respectively. Additionally, a few other studies have reported the application of machine vision approaches using different machine learning and deep learning methodologies for detecting and classifying different flower nurseries [71,80,81,82,83,84].

**Table 4:** Summary of studies reported for plant biometric measurements.

Crops	Sensor Type	Model	Performance	References
Seven different plant cultivars–container	RGB camera	Color co-occurrence matrices (intensity, saturation, and hue)	Overall classification accuracy of 91%	Shearer and Holmes [74]
Perennial peanut and Fire chief arborvitae–container	RGB camera	Vegetation index thresholding and the support vector machine (SVM)	Accuracy of more than 94%	She et al. [75]
Fire Chief arborvitae–container	UAS-based RGB camera	Custom counting algorithm	Counting error on gravel and black fabric of 8% and 2%, respectively	Leiva et al. [76]
Spruce, Mongolian scotch pine, Manchurian ash–field	RGB-Depth camera	YoloV4 with Ghostnet	Accuracy of more than 92% in both counts and height measurements	Yuan et al. [77]
Eleven different tree nurseries–field	UAS-based Multispectral camera	Grey Level Co-occurrence Matrix for texture images; Maximum Likelihood algorithm, and Principal Component Analysis	Accuracy of 87%, depending on components reduction on spectral camera	Gini et al. [78]
Six different species–container	LiDAR sensor	Logistic regression functions, support vector machines (SVM)	Accuracy greater than 98%	Weiss et al. [79]
Almond tree nursery	LiDAR and light curtain sensors	Custom segmentation and thresholding algorithm	Tree detection acc of 95.7% (LiDAR) and 99.48% (light curtain sensors); Dead/alive tree detection acc of 93.75% (LiDAR) and 94.16% (light curtain sensors)	Garrido et al. [73]
Flower-Field	RGB camera	ResNet18, ResNet50, and DenseNet121	Accuracy of 91.88%, 97.34%, and 99.82% respectively	Zhang et al. [71]
Flower-Field	RGB camera	DenseNet121	Accuracy of 98.6% for 50 epochs	Alipour et al. [80]
Flower-Field	RGB camera	CNN, VGG16, MobileNet2, and Resnet50	Test accuracy: 91%, 89.35%, 92.12%, 71.75%, respectively	Narvekar and Rao [83]
Flower-Field	RGB camera	Custom and Inception v3	Accuracy of 83% and 99%, respectively	Dharwadkar et al. [81]
Flower-Field	RGB camera	Naive Bayes (NB), Generalized Linear Model (GLM), Multilayer Perceptron (MP), Decision Tree (DT), Random Forest (RF), Gradient Boosted Trees (GBT), and Support Vector Machine (SVM)	RF is the best-performing model, with an accuracy of 78.5%.	Malik et al. [82]
Flower-Field	RGB camera	Viola-Jones object detection and normalized cross-correlation algorithm	Classification accuracy of more than 99% with <0.5 s processing time	Soleimanipour and Chegini [84]

Nursery crop management is time-consuming and labor-intensive, bringing a great need for automation, especially for large nursery production areas. Sensing-based plant biometrics, identification, and recognition are promising but challenging tasks. The rapid advancements in sensing, computation, artificial Intelligence (AI), and data analytics have allowed more detailed investigations in this domain. Research has been reported to identify tree species for management operations and counting plants for inventory control using different types of sensors, including RGB, multispectral, LiDAR, etc. A few recent studies have utilized state-of-art deep learning techniques for nursery plant classification; however, more efforts are needed to facilitate the growers’ use of such techniques for the profitability and sustainability of the nursery industry.

### 2.5. Other Significant Works

The economics of production practices associated with fertilizer inputs, pest control needs, and labor requirements affect the nursery industry. Most nursery production operations are labor intensive. According to Gunjal et al. [85], labor accounts for 70% of the costs for nursery production. Though a few operations in nursery production have been mechanized, many others have not been automated. Advanced sensing and mechanization/automation could reduce resource consumption and labor dependence [73]. In this context, the ornamental nursery industry has witnessed some progress in different sensing, automation, and robotic applications. Table 5 presents the summary of works related to other sensing and automation applications for nursery crop production. Li et al. [86] developed a trimming robot for ornamental plants. The design includes a knife system and a rotary base, allowing the knife to rotate 360 degrees to cut the plants into the desired shape. The robot was tested for five different nursery plant species (*Aglaia odorata*, *Murraya exotica*, *Camellia oleifera*, *Osmanthus fragrans*, and *Radermachera sinica*), and results indicated that the overall performance was above 93% with the time taken as 8.89 s. Zhang et al. [87] developed a path-planning scheme for a watering robot for containerized ornamental nursery plants. The authors optimized the robot’s path planning using a genetic algorithm with neighbor exchanging to test different watering strategies, and achieved promising results in terms of water savings. Sharma and Borse [88] developed an autonomous mobile robot to carry out different production operations in the nursery. The robot featured multiple sensor modules, including camera and climate monitoring, to perform real-time growth monitoring, disease detection, and the spraying of fertilizer, pesticide, and water. The platform was also equipped with a Zigbee communication framework to transmit the sensed data to the central control system. The system achieved the desired results for disease detection and growth monitoring; however, no technical details are provided. Similarly, a conceptual design of a cable-driven parallel robot (CDPR) to perform different operations, including seeding, weeding, and nutrition monitoring for plant nurseries has been presented [89]. The authors performed the operational and path planning simulation to execute seeding and weeding operations. Additionally, a pretrained VGG16 model was used for weed identification, and results showed promise, with an accuracy of 96.29% achieved during testing. Despite some progress, the status of research-based findings for robotic applications in the nursery industry lags far behind its contemporary industries.

## 3. Future Prospects/Directions

### 3.1. Advanced Camera Sensor Applications

#### 3.1.1. ToF, LiDAR, and 3D Sensors Applications

Advanced sensing technologies, such as depth cameras, time-of-flight (ToF) cameras, and multispectral and hyperspectral cameras, have been widely used in different agricultural applications. Kim et al. [90] implemented a binocular stereo-vision camera incorporated with a single-board computer for estimating crop height. Authors successfully estimated heights for Chinese cabbage, potato, sesame, radish, and soybean crops with a less than 5% of error in field conditions. Wang et al. [91] developed a ground-based remote imaging system comprised of an ultrasonic sensor, a LiDAR sensor, a Kinect camera, an imaging array of four digital cameras, and a custom-developed gimble and camera, respectively, for estimating sorghum plant height at plot level. The author observed that an ultrasonic sensor, a LiDAR sensor, and a Kinect camera resulted in strong correlations (r ≥ 0.90) between automatic and manual measurements for plant height estimation. The study concluded that the ground-based image acquisition system resulted in a comparatively higher correlation between automatic and manual measurements compared to the remote imaging system. They recommended LiDAR combined with high-resolution camera array technology, which can be an ideal methodology for measuring plant height effectively. The 3D/Depth cameras have found widespread usage in agriculture for a variety of purposes, including but not limited to yield estimation [92], plant phenotyping [93], and disease detection [94].

A vision-based under-canopy navigation and mapping system for corn and sorghum was developed by Gai et al. [95] using a ToF camera combined with a field robot, PhenoBot 3.0. They implemented linear programming techniques and developed a novel algorithm for reliable crop row detection and navigation. The developed system achieved mean absolute errors (MAE) of 3.4 cm and 3.6 cm in fields of corn and sorghum, respectively. Similarly, Gongal et al. [96] fused a color charge coupled device (CCD) camera and a ToF sensor to estimate apple fruit size under controlled lighting conditions. The developed system estimated apple fruit size with an accuracy of 84.8% based on pixel size. A few of the most significant applications for ToF cameras in agriculture are plant height estimation [97,98], 3D reconstruction of the plant [99], 3D plant morphology [100], palm bunch grading [101], and so on.

#### 3.1.2. Spectral Sensor Applications

Cao et al. [102] developed a nitrogen monitoring system for tea plants using multispectral (wavelengths: 475 nm, 560 nm, 668 nm, 717 nm, and 840 nm) and hyperspectral imaging systems. They fused data after preprocessing, which included multispectral image registration, calibration, information extraction and selection, and hyperspectral wavelength selection. After filtering the fused data, they feed them to regression models, including PLS regression, random forest regression (RFR), and support vector machine regression (SVR), to predict the nitrogen content of tea leaves. The support vector machine regression outperformed other models and achieved R^2^ (coefficient of determination) and root mean square error values of ~0.92 and ~0.06, respectively. Another researcher, Chandel et al. [103], also used simple linear regression models (LRs) to experiment on characterizing Alfalfa (*Medicago sativa* L.) crop vigor and yield by combining multispectral (465–860 nm) and thermal infrared (11,000 ± 3000 nm) image data collected from unmanned aerial vehicles. The model MLR-4 outperformed other models and achieved an R^2^ of 0.64. 

The aforementioned studies offer compelling evidence of increased success rates for agricultural applications of cutting-edge sensors, which suggest prospective uses for ornamental nursery crops. The advanced sensors can operate successfully in both indoor and outdoor environments. Therefore, in the future, automated systems for ornamental nursery corps can be developed using sophisticated camera sensors like 3D or depth cameras, ToF, multispectral, and hyperspectral.

### 3.2. Enhanced Deep Network Applications

Due to the extraordinary ability to generate synthetic datasets with the same properties as training datasets, advanced computer vision-based techniques such as generative adversarial networks (GANs) and transformers are overtaking photometric and geometric-based augmentation approaches in a variety of agricultural problems. Abbas et al. [104] proposed a tomato plant disease detection system using a publicly available plant village tomato leaf dataset. The authors augmented the dataset using a conditional generative adversarial network (C-GAN) and fed the data to a pre-trained DenseNet network. The network successfully predicted tomato leaf diseases from healthy leaves and achieved an accuracy of 97.11%. The augmentation of the tomato leaf dataset improved the DenseNet network’s prediction by 2.77% compared to the accuracy of the original plant village tomato leaf dataset. Xiao et al. [105] implemented Texture Reconstruction Loss CycleGAN (TRL-GAN) to produce phenotypic data for the citrus greening disease and improve classification networks for the detection of diseased leaves. The authors observed that the TRL-GAN based method improved accuracy by 2.76% compared to the baseline model and 1.04% compared to the traditional augmentation methods (rotation and stretching). Zhang et al. [106] combined hyperspectral imaging with generative adversarial networks (DCGAN, CGAN) to expand the original dataset. They also observed that expansion of the dataset using GANs would improve the accuracy of k-nearest neighbor (kNN), SVM, and RF for haploid maize kernel classification by 12%, 20%, and 12%, respectively, compared to baseline models.

Data enlargement using GANs allows for the development of detection, classification, and prediction models with less data on ornamental nursery crop images, which increases the model’s resilience in varying conditions and improves performances or accuracies. The augmented data can be incredibly useful when developing machine vision-based systems for nursery crops, such as leaf classification and disease assessment systems. Robots may be trained in a simulated environment using the data produced by GANs.

### 3.3. Edge-AI Applications

Embedded platforms combined with hardware accelerators and artificial intelligence-based sensing technology, called Edge Artificial Intelligence (Edge-AI), have made quick responses with low latency possible over cloud-based solutions. This technique has been adopted in different agricultural applications in recent years. Mazzia et al. [107] developed a real-time apple detection system using an Edge-AI technology. They implemented YOLOv3-Tiny algorithms on three different embedded platforms, including Raspberry Pi 3 B+ with Intel Movidius Neural Computing Stick (NCS), Nvidia’s Jetson Nano and Jetson AGX Xavier, and successfully detected apples in an orchard. Their system achieved an accuracy of 83.64% with a data processing speed up to 30 frames per second (fps) in complex situations. Zhang et al. [108] implemented YOLOv4-Tiny networks combined with improved cross stage partial networks (CSPNet) in the backbone for strawberry detection and implemented a developed model on the embedded platform Jetson Nano (NVIDIA Corporation, Santa Clara, CA, USA). Their optimized model (RTSD-Net) with TensorRT achieved about 25.20 fps and performed 15% faster than the original YOLOv4-tiny model on Jetson Nano without significant loss of accuracy. Other promising applications of Edge-AI are air temperature forecasting [109], environment monitoring [110], autonomous navigation systems [111] and so on. 

Edge-AI technology can potentially be applied for weeding, spraying, and robot navigation in ornament nursery crop production. Weed maps generated by UAVs may be combined into autonomous robots for site-specific weed management and pesticide applications in the field. Embedded hardwire (Raspberry Pi, Jetson Nano, and Jetson TX2) paired with sensors (color camera, depth camera), and AI may be implemented to develop Edge-AI technology for ornamental nursery crops. Vision-based robots using Edge-AI technology can be an aid to robot navigation for accomplishing site-specific applications in nursery crops.

### 3.4. Radio Frequency Identification Tagging Applications

Radio frequency identification (RFID) technology has become popular in different fields of agriculture, including soil environment monitoring, soil moisture monitoring, soil solarization, and automation in irrigation. Deng et al. [112] designed and developed a novel system that integrates an RFID sensor with LoRa to provide a low-cost, low-power, and efficient soil environment monitoring solution. The authors embedded RFID tags at 60 cm into the soil; the tags can communicate with the monitoring center through radio communication (LoRa) placed in the patrol car. Their system would be able to establish communication within a range of 1.3 m without compromising relative measurement errors (temperature: 1.5% and soil moisture content: 1.0%). The study achieved a higher communication rate (above 90%) at a patrol speed of 33 kmh^−1^. Luvisi et al. [113] developed a system that monitors different types of soil solarization (sandy, loam, and clay soils) using an RFID sensor and biodegradable films. They placed soil sensors at different depths (5 and 10 cm) along with a soil profile at different soil moisture holding capacities (10%, 50%, and 90%) and measured the effect of soil solarization treatment. In the second and third weeks of treatment, they found that the maximum soil temperature at depths of 5 and 10 cm increased to 9–13 °C and 11–14 °C, respectively. They also found that the method was 90% reliable. Vellidis et al. [114] implemented soil moisture sensors (Watermark^®^ granular resistive type) and thermocouple temperature sensors coupled with RFID tags (WhereNet^®^, Santa Clara, CA, USA) for developing sensor nodes to automate irrigation schedules for cotton crops. The nodes were connected to a laptop computer via wireless communication. The developed system contained an array of sensors, and data obtained from the sensors could assist in decision-making and scheduling irrigation for the cotton field. Several researchers also contributed to RFID-based soil moisture sensor developments [115,116,117,118,119]. RFID-based sensors also have other applications in agriculture, including tracking plants in pots in greenhouses [120], tracing food quality [121], and monitoring livestock [122]. 

The above studies and their success rates clearly show the potential of using RFID-based sensors in ornamental nursery crop applications. The potential applications of RFID-based sensors for nursery crops include soil environment monitoring, soil solarization, and automating irrigation scheduling, in indoor or field conditions. The RFID tags can be used in conjunction with soil monitoring sensors such as soil, moisture, soil micronutrient, gas, etc. to build sensor nodes and receive in-field data through wireless communications. Readings from sensor nodes may be used with machine learning and deep learning to make decisions in various field management operations.

### 3.5. Integrated Robotics Applications

Robots integrated with computer vision have been widely adopted in many areas of agriculture, such as plant detection and mapping, fruit detection and localizations, robot-based harvesting, navigation, and obstacle detection systems. Weiss and Biber [123] developed a ground-based robot for maize plant recognition, mapping, and navigation using a 3D LiDAR sensor-based micro-electro-mechanical system (FX6 3D LiDAR). The robot was constructed using modeled artificial maize plants and tested on a small corn field. The designed robot achieved detection and mapping accuracy of around 60%–70%. They measured a greater localization deviation in the direction of the row, measuring 1–2 cm. Ge et al. [124] developed a strawberry fruit localization method using a strawberry harvesting robot with an RGB-D camera. The authors implemented a convolutional neural network (i.e., Mask-RCNN) on RGB images for strawberry fruit segmentation and combined depth values to obtain 3D points of fruits. The 3D point was then used to obtain fruit localization using the shape completion method. The system achieved a minimum center deviation of 6.9 mm between ground truths and automated measurements. Skoczeń et al. [125] also proposed a similar approach to develop an automatic obstacle-detection robot. They implemented an RGB-D camera (Intel RealSense D435i) for robot vision, reached obstacle segmentation accuracy of 98.11%, and obtained a depth measurement error of 38 cm. 

Ji et al. [126] developed a machine vision algorithm for a green pepper harvesting robot. The contrast values of images obtained by the camera (MX808) for various light conditions (normal, weak, and strong light) were then increased to make the green pepper stand out from the background leaf. The energy-driven sampling (SEEDS) algorithm is then fed the improved images to build super pixel blocks. The manifold ranking (MR) algorithm, the CART classifier, and the conditional random field (CRF) algorithm were used to recognize green pepper from super pixel blocks, followed by morphological processing. Classifiers were evaluated on 500 images obtained from different lighting conditions. The algorithm manifold ranking outperformed other classifiers and achieved an accuracy of 83.6%; it took 116 milliseconds to run the entire evaluation on Intel Core (TM) i5-4210U CPU (2.80 GHz, 8 GB). Gai et al. [127] developed a cherry fruit detection system using a high-resolution Sony DSC-HX400 camera combined with a YOLO-V4 Dense Model network. The study compared the developed algorithm’s accuracy with the base model, YOLO-V3-dense, and YOLO-V4 and observed an improved detection rate (F1 scores: 94.70%). YOLO-V4 Dense Model took 0.467 s on an Intel Core (TM) i7-7700 CPU (3.60 GHz, 4 GB) with a Tesla V100 GPU for processing an image of 1280 by 800 pixels. They found robotic intelligent picking is possible using the developed system. Jia et al. [128] also developed a robot vision using a high-resolution camera (6000 × 4000 pixel) for an apple harvesting robot using an optimized Mask R-CNN. The developed system achieved a high rate of detection (precision: 97.31%; recall: 95.70%).

The development of robot vision using high-resolution camera sensors combined with deep learning techniques can be adopted to develop ornamental crop management robots. Applications, such as spraying, weeding, soil sampling, and digging could be effectively solved, enabling different operations in nursery crops. Robot vision combined with machine and deep learning may also be implemented in nurseries for plant counting, stem counting, and other essential tasks.

## 4. Discussion and Conclusions

The ornamental crop industry in the U.S. depends largely on agricultural workers. Sensing and automation technologies offer a huge potential to reduce labor dependency and ensure the efficient use of resources required by the ornamental industry. In turn, the information in this article can aid the nursery industry in knowing about the specific area where technological development takes place and what those technologies are, and in considering what types of sensors, algorithms and tools are advantageous to develop effective technologies in different production operations.

Current sensing and automation technology usage varies by production operations. For instance, smart irrigation has primarily relied on soil moisture sensors, and stress detection has largely depended on camera sensors. Despite the fact that not many studies have used IoT or Edge-AI-based IoT systems, these could be potential technologies for automating irrigation operations for ornamental crops. The Edge-AI-based systems and AI-of-things (AIoT) are relatively new concepts in agricultural applications, and successes in other cropping systems have shown promise for the ornamental nursery industry. Similar to irrigation, a very limited number of studies have been conducted for ornamental plant stress detection. One important fact regarding stresses is that they have to be detected early to minimize their effect on crops. Spectral cameras, including hyperspectral and multispectral devices, are the two sensors currently being used to detect stresses at the asymptomatic stage. However, the major challenge of detecting plant stresses is to detect them in real-time field conditions. Researchers have been trying to address challenges such as illumination variations, data processing speed, and environmental factors to make a viable system for real-time applications. More efforts are required, though, especially for the hyperspectral system, due to its slow data processing issues. Fluorescence sensors are another spectral technology that has not been explored much for ornamental crops, one which can provide improved spectroscopy data and can be useful for early plant stress detection. LiDAR is one of the powerful tools that can be used to accurately measure plant biometric information (plant height, width, canopy volume and density, etc.) to develop a smart or variable-rate spraying system. However, this tool cannot be used for spot spraying operations for disease management because the LiDAR sensor can only provide point cloud information (unlike cameras, it does not provide any color information). Integrated LiDAR and camera systems could potentially be tools for smart spraying systems for ornamental nursery crop production. The advantages and disadvantages of different sensors are presented in Table 6.

Surprisingly, very few applications have been noticed for UAVs in ornamental crops, despite extensive implications these days in the agronomic, tree fruit and row crops. The low manufacturing cost and fast operation speed have opened up further research opportunities for UAVs. UAVs are becoming an essential part of remote sensing and can be an effective tool for ornamental plant stress detection and monitoring crop growth and development. The UAVs bring advantages over ground-based systems, such as their flexibility in capturing ultra-high spatial and temporal resolution data at any terrain conditions, and they require less time to collect data. However, developing manipulation systems for UAVs that can act with precision in fields is a challenging task requiring extensive investigations. The coordination between UAVs and ground-based systems has been receiving increasing attention in recent years, and has the potential to benefit the ornamental crop industry for site-specific management. Calibrating sensors is essential to reduce variability when multiple sensors are involved in a particular crop management operation.

Recent advances in deep learning models (e.g., CNNs, GANs, transformers) have contributed significantly to different industries, including agriculture, but ornamental crops remain at the bottom user of these impressive innovations. These models can help predict stress, pest pressure, growth, yield, etc. RFID, a new crop tracking technology, can increase production operations’ efficacy and help nurseries to reduce the burden for growers or laborers by automating the inspections and recording accurate ornamental crop data instantly. Agricultural robotics is another critical area that can benefit the ornamental crop industry enormously. Currently, the agricultural workforce conducts most production operations, such as planting, pruning/shape forming, weeding, disease monitoring, and harvesting. These operations are vastly labor-intensive and cost a large portion of production expenses. Autonomous robotic systems can replace the humans conducting these operations. The systems will reduce time and production expenses in the long run. The ornamental industry lacks automation/robotic technologies; therefore, significant research needs to be done on these topics to develop some implementable robotic systems.

As the majority of the ornamental crop farms are not so large compared to other major cropping industries, adopting advanced sensing and automation technologies would be a major challenge due to the initial high investment. Integrated multipurpose automated technologies will be helpful for this purpose. For instance, when a particular automated system can work for multiple operations (e.g., planting, pruning, and harvesting) for ornamental crops by replacing a few parts of the system, growers would be interested in buying and adopting those multipurpose systems. Researchers and manufacturers need to consider these points while working on or developing technologies for the ornamental nursery crop industry.

Although not much progress in sensing and automation technologies has been observed for ornamental nursery crop production, a few mechanized systems are available for commercial scales. These include mixing systems to mix substrate or soil, potting systems to fill containers, tray filling systems to fill trays, planters to plant nursery liners in containers, seeding systems to sow and space out seeds on pots or containers, etc. Pack Manufacturing (http://packmfg.com/) (Pack Manufacturing Inc., McMinnville, TN, USA) is a leading company in the sale of these mechanized systems.

A vital challenge in technology development for ornamental nursery crops is the substantial number of available plant species. Various ornamental plants have different morphologies, characteristics, canopy structures, and growth requirements. It is necessary to understand the types of plants grown and their production requirements to align the sensing and automation technologies with the production needs to facilitate industry operations.

## Figures and Tables

**Table 5:** Summary of works related to nursery production in other remaining areas.

Crops	Nursery Types	Specifications	Performance	References
Multiple species of nursery plants	Container grown	Genetic algorithm for optimized path planning	Reduced water consumption; the optimal path for watering	Zhang et al. [87]
Five different plant species	Container grown	Integrated knife and rotary base for trimming	Overall performance was more than 93%; time: 8.89s	Li et al. [86]
Unspecified	Field grown	Algorithm: Support Vector Machine (SVM)	High accuracy for disease identification and growth monitoring	Sharma and Borse [88]
Unspecified	Field grown	Cable-driven manipulator; pre-trained VGG16 for vision system	Weed detection accuracy of 96.29%; accurate trajectory planning in simulation	Prabha et al. [89]

**Table 6:** Advantages and disadvantages of different sensors for ornamental crops.

Sensor Types	Advantages	Disadvantages
Image sensors (RGB camera, multispectral, hyperspectral, etc.)	Capable of detecting diseases, stresses, and weeds in ornamental crops; Ability to provide 3D information for pruning, shape forming, weed management and other operations; Potential to replace humans for crop monitoring using drones.	Sensitive to weather conditions, especially illumination conditions; Some image sensors, such as hyperspectral, are expensive.
Range sensors (LiDAR, ultrasonic, etc.)	Sensors, especially LiDAR, not affected by environmental conditions; Plant biometrics (height, canopy volume, density, leaf area index, etc.) accurately determined; High speed of canopy parameter measurement.	Cannot provide detailed crop information (only provide point cloud data); Vibration during operation can significantly affect the sensor performance.
Infrared sensors (temperature sensors)	Provide crop temperature information; Very important to detect crop drought stress for ornamental crops; Capable of detecting imported fire ant colonies in ornamental crop fields.	Cannot detect multiple objects with a small temperature differences; Fairly expensive.
Volumetric sensors (soil moisture sensors)	A simple method of measurement; Can directly measure the amount of water in the soil; Delivers the results immediately; Low in cost.	Requires initial evaluation of site-specific conditions before selecting sensors; Accuracy is low in sandy soils due to large particles.
